# Sepsis carries a high mortality among hospitalised adults in Malawi in the era of antiretroviral therapy scale-up: A longitudinal cohort study

**DOI:** 10.1016/j.jinf.2014.07.004

**Published:** 2015-01

**Authors:** Peter I. Waitt, Mavuto Mukaka, Patrick Goodson, Felanji D. SimuKonda, Catriona J. Waitt, Nick Feasey, Theresa J. Allain, Paul Downie, Robert S. Heyderman

**Affiliations:** a Department of Medicine, College of Medicine, University of Malawi, Malawi; b Malawi-Liverpool-Wellcome Clinical Research Programme, University of Malawi College of Medicine, Blantyre, Malawi; c Department of Anaesthetics, College of Medicine, University of Malawi, Malawi

**Keywords:** Sepsis, HIV, Bacteraemia, Antiretroviral therapy, Africa, Adults

## Abstract

**Objective:**

To assess mortality risk among adults presenting to an African teaching hospital with sepsis and severe sepsis in a setting of high HIV prevalence and widespread ART uptake.

**Methods:**

Prospective cohort study of adults (age ≥16 years) admitted with clinical suspicion of severe infection between November 2008 and January 2009 to Queen Elizabeth Central Hospital, a 1250-bed government-funded hospital in Blantyre, Malawi. Demographic, clinical and laboratory information, including blood and cerebrospinal fluid cultures were obtained on admission.

**Results:**

Data from 213 patients (181 with sepsis and 32 with severe sepsis; M:F = 2:3) were analysed. 161 (75.6%) patients were HIV-positive. Overall mortality was 22%, rising to 50% amongst patients with severe sepsis. The mortality of all sepsis patients commenced on antiretroviral therapy (ART) within 90 days was 11/28 (39.3%) compared with 7/42 (16.7%) among all sepsis patients on ART for greater than 90 days (*p* = 0.050). Independent associations with death were hypoxia (OR = 2.4; 95% CI, 1.1–5.1) and systolic hypotension (OR 7.0; 95% CI: 2.4–20.4).

**Conclusions:**

Sepsis and severe sepsis carry high mortality among hospitalised adults in Malawi. Measures to reduce this, including early identification and targeted intervention in high-risk patients, especially HIV-positive individuals recently commenced on ART, are urgently required.

## Background

Severe sepsis is a leading non-cardiovascular cause of death in critically ill patients worldwide, with 90% of deaths from pneumonia, meningitis and other infections occurring in low-resource settings. In countries such as Malawi, where there is a high burden of HIV-related disease,^1^ sepsis is thought to be a major killer. However, despite numerous studies of microbiologically-proven bloodstream infections (BSI) in sub-Saharan Africa (SSA),^2,3^ few have sought to systematically evaluate patients against internationally defined criteria for sepsis in such settings.^4^

Case definitions for sepsis, severe sepsis and systemic inflammatory response syndrome (SIRS) were developed in 1992^5^ and with refinements in 2002,^6^ 2008^7^ and 2013.^8^ The Surviving Sepsis Campaign, recommending ‘bundles’ of early, specific interventions has led to demonstrable improvements in clinical outcomes in severe sepsis in well-resourced settings.^9^ However, although early identification and treatment of sepsis in low-income countries have been highlighted as essential components of good clinical care by the World Health Organisation (WHO),^10^ lack of data regarding the clinical manifestations of severe sepsis from many such countries renders it problematic to derive evidence-based guidelines.^11–13^ Differences in age range, spectrum of aetiology, and co-morbidities such as HIV, TB and malaria makes extrapolation of data from high/middle-income to low income countries unreliable. Furthermore, resource limitations are a significant constraint to implementing even simple interventions.^11^

This study therefore aimed to assess the risk of death among adult medical patients presenting to hospital with syndromically defined sepsis and severe sepsis in the context of a low income African setting with high HIV prevalence. Furthermore, we have investigated the impact of ART on clinical outcomes from sepsis and severe sepsis in this environment and sought to identify additional simple physiological assessments that could be used to identify high risk patients in whom interventional trials are warranted.

## Methods

### Setting

Queen Elizabeth Central Hospital (QECH) is a 1250-bed government-funded teaching hospital providing secondary and tertiary care, free of at the point of care to the patient. QECH serves a population of approximately one million including the city of Blantyre, the surrounding townships, and outlying villages. At QECH, measurement of central venous pressure, blood gas analysis and urine output are logistically difficult and rarely performed. Vasopressors and inotropes are unavailable on the medical wards. High-flow oxygen from cylinders is unavailable, therefore oxygen therapy is provided through oxygen concentrators when available. Patients with severe sepsis and septic shock are rarely admitted to the QECH intensive care unit (ICU) because of high bed occupancy and perceived futility for such patients. On the adult medical wards, two nurses are typically responsible for between 60 and 90 patients (greater than 100% bed occupancy is common).

### Patients

Consecutive adults (age ≥16 years) with a clinical suspicion of severe infection (as determined by the admitting clinician) admitted to the Department of Adult Internal Medicine at QECH, between November 2008 and January 2009 were prospectively recruited following informed consent from the patient or their guardian. Enrolment, assessment and follow-up were conducted by a dedicated research team and recruitment did not take place at weekends or outside routine working hours on weekdays due to staffing constraints. Patients were excluded from enrolment if they had been hospitalised or received antibiotics in the preceding two weeks, or if it was not possible to obtain written consent from the patient (e.g. an obtunded patient with no guardian available). Patient demographics, clinical and laboratory characteristics were recorded on a standardised assessment form. Follow-up was to hospital discharge or in-hospital death.

#### Case definitions

Sepsis and severe sepsis were identified using modified standard criteria as set out in Table 1a and 1b.^4,6^ Due to resource constraints, markers of severe sepsis were limited to those which could be assessed clinically or through simple laboratory tests. Capillary refill time is recognised as a surrogate for end tissue perfusion.^14^ Oxygen saturations have been used as surrogate for partial pressure of oxygen. Thrombocytopenia was not used as a marker of severe sepsis in HIV-infected individuals.^15,16^ Tuberculosis was suspected in patients who failed to respond to antibiotics for presumed pneumonia or in whom there were suspicious CXR changes; investigation and treatment were instigated at the discretion of the responsible clinician in accordance with national guidelines.^17^

All patients were screened for malaria, and all patients with a positive malaria film received either oral lumefantrine-arthemeter or intravenous quinine according to national guidelines.

Given its unpredictability and the very low nursing coverage available, the mode of death could not be captured. Autopsies were not routinely available. Retrospective chart review was not feasible because patient notes are frequently unavailable following discharge and do not contain the information necessary for a study of this nature.

### Laboratory investigations

Patients had 5–10 mL of blood drawn for aerobic culture in an automated system (BacT/ALERT, Bio-Merieux). A full blood count (Coulter Hmx Haematology Analyzer), malaria thick film and HIV testing using Determine™ HIV 1/2 kit (Abbott Diagnostic Division) and Unigold™ HIV 1/2 kit (Trinity Biotech Inc.), according to manufacturers' instructions, were performed in all patients. Lumbar punctures (LP) and standard CSF analysis were performed when there was a clinical suspicion of meningitis. Identification of blood and CSF isolates was performed using standard methods with external quality control (United Kingdom National External Quality Assessment Service).^18–20^ Coagulase negative *Staphylococci*, Diptheroids, *Micrococcus spp* and *Bacillus spp* other than *anthracis* were considered as contaminants. Mycobacterial blood cultures were not performed due to resource constraints. Additional investigations were undertaken by the responsible medical team as considered clinically indicated. CD4 counts were not routinely available.

### Statistical analysis

Statistical analyses were performed using STATA for windows software (version SE/11; 4905; Stata corp; College Station, Texas 77845 USA). Statistical tests were performed at 5% significance level. Descriptive analysis of baseline variables was performed to summarize patient characteristics. *T*-tests compared means of normally distributed and Mann–Whitney *U* tests compared medians (the distribution) of the variables with skewed distributions respectively between the sepsis and severe sepsis groups. Fisher's exact test was used to assess an association between a binary variable and diagnosis (whether patient had sepsis or severe sepsis), with *p* values of less than 0.05 considered significant. Fisher's exact test was preferred to the Pearson's Chi-square tests for associations because it has superior statistical properties when the numbers are small as is the case in this study. Time to event, where time was admission duration and event was death, was modelled using survival methods such Kaplan Meier plots, log-lank tests and the Cox proportional hazards regression models. Kaplan–Meier (KM) survival curves were compared with the log-rank test. Patients lost to follow-up before discharge were censored at their last known assessment. Univariate logistic regression identified variables associated with outcome (death), with subsequent multivariate logistic regression to obtain adjusted estimates. A stepwise variable selection procedure was used to find independent predictors of outcome (death) with p-to-enter of 0.05 or less, and p-to-remove of 0.15. The 95% confidences intervals were obtained where applicable. A logistic regression was also used to identify factors associated with sepsis.

In addition, KM curves were plotted to compare time to death from time of admission between HIV positive and HIV negative patients. The Cox proportional hazards regression model was fitted to obtain the hazard ratios, 95% confidence intervals (CI) and corresponding *p*-values. KM plots were also plotted for the HIV subset comparing the survival probabilities by ART status.

### Ethics statement

Ethical approval for the study was prospectively obtained from the College of Medicine Research and Ethics Committee, University of Malawi (COMREC no P.05/08/667).

## Results

### Patient characteristics

**Figure 1:**
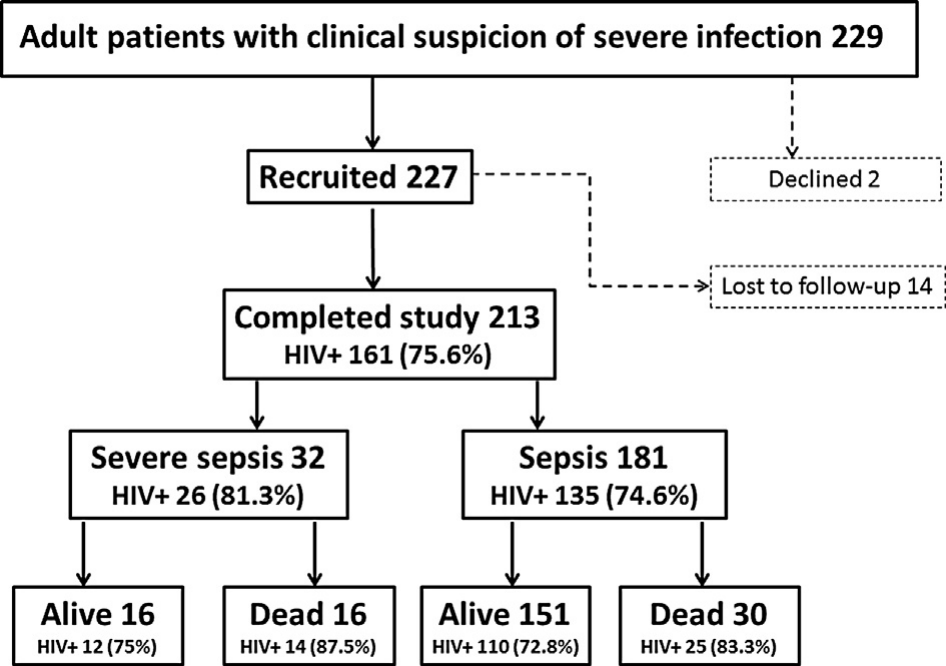
Patient recruitment.

Two hundred and twenty nine patients fulfilled the sepsis criteria including 32 who fulfilled the severe sepsis criteria. This represents 18% of the 1250 patients who were admitted to the adult medical wards during that time period (hospital data). Informed consent could not be obtained for 12 moribund patients who may have met inclusion criteria, and these do not feature in subsequent analysis. Two hundred and twenty seven were enrolled (Fig. 1). Fourteen patients were lost to follow-up during their inpatient stay due to premature self- or family initiated discharge. Analysis was conducted on the remaining 213 (93.0%) patients (181 with sepsis and 32 with severe sepsis). Intravenous ceftriaxone was used as empirical first line therapy, with no differences in antibiotic usage between patients who died and survivors. No patients were admitted with indwelling intravascular or ureteric catheters. There were no patients on treatment for chronic renal, lung or cardiovascular disease.

Descriptive demographic, HIV and clinical characteristics of the cohort are summarised in Table 2. The median age was 30 years (IQR 25–39), with no significant difference between those with sepsis and severe sepsis. 76% were HIV infected and of these, 70 out of the 161 HIV infected, 43.5% were on ART. The majority of HIV-infected patients (55%) were unaware of their HIV status prior to enrolment in the study (this is typical for all patients admitted to these wards). As anticipated, features of systemic inflammatory response and impaired tissue perfusion such as pulse rate, respiratory rate, systolic blood pressure (SBP), Glasgow Coma Scale (GCS), capillary refill time and oxygen saturation were generally more abnormal amongst the severe sepsis compared with the sepsis patients (Table 2). The lung (based on clinical symptoms and signs suggestive of respiratory tract infection) was the most common focus of presumed infection but radiological confirmation by either X-ray or ultrasound was not always available. This was followed by sepsis of unknown source (49 patients) and meningitis (insert numbers here) identified by lumbar puncture with a raised CSF white cell count (7 patients were culture confirmed). Forty patients (18.8% of the cohort) were clinically suspected of having tuberculosis on the basis of the criteria described above and commenced on treatment, microbiological confirmation was obtained in 14 (35% of TB suspects). There were no patients in whom immune reconstitution inflammatory syndrome (IRIS) was suspected clinically. Unmasking of occult cryptococcal disease was not detected but could not be excluded.

As shown in Table 3, HIV negative patients with severe sepsis had significantly lower median platelet counts than those with sepsis (79 × 10^9^/L [IQR 43–168] vs 153 × 10^9^/L [IQR 98–240] respectively; *p* < 0.001). Bacteraemia was identified in 32 (15.0%) of all study patients and eight of the 32 (25.0%) with severe sepsis. There were 7/213 (3.3%) patients who had culture proven meningitis in addition to sepsis or severe sepsis. Bacteraemic patients had a mortality of 25% compared with 21% in those with a negative blood culture (*p* = 0.600). The profile of organisms was as anticipated from previous studies in Blantyre and other similar settings, with non-typhoidal Salmonellae (NTS) and *Streptococcus pneumoniae* predominating (Table 3).^2,21^ Resistance of these common isolates to ceftriaxone was not observed. Twenty six (12%) patients had a positive malaria film (1/26 [3.8%] with severe sepsis), but no deaths were attributed to malaria as no patients with a positive malaria film died … It was thought clinically that this was incidental asymptomatic parasitaemia, due to both the low levels of parasitaemia identified and the fact that adults in this region have usually developed partial immunity, with severe malaria being predominantly a disease of childhood.^18^

### Mortality

**Figure 2:**
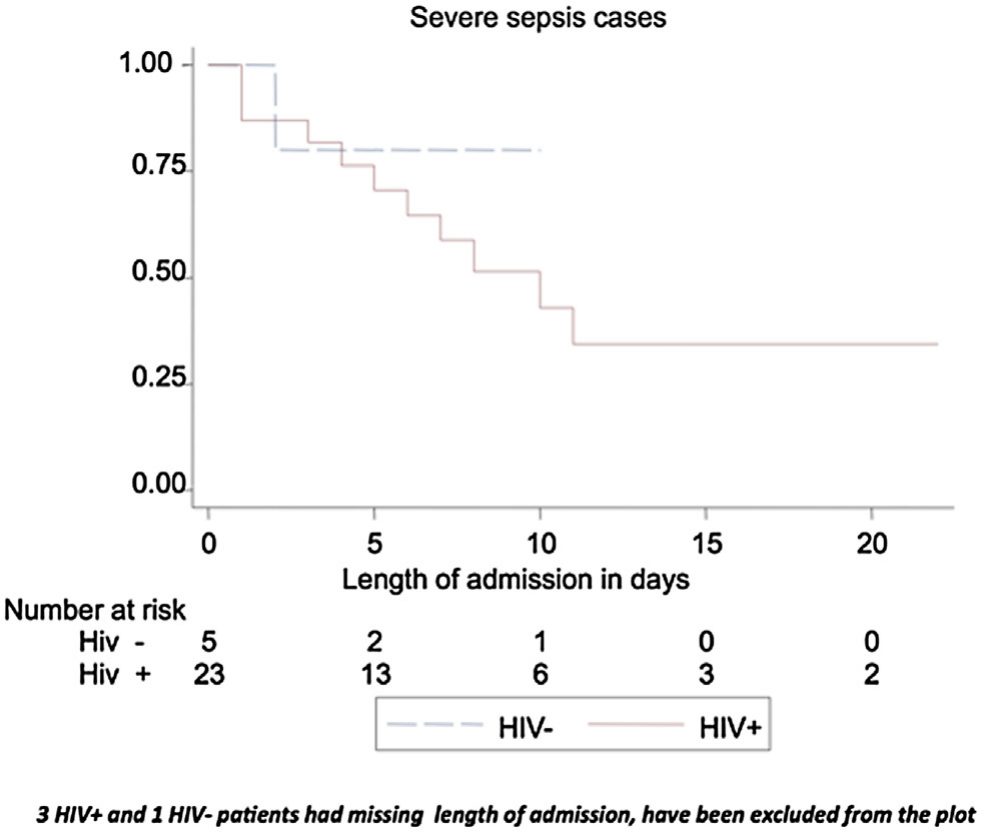
Kaplan–Meier survival curve of patients with confirmed severe sepsis. Comparison between HIV positive and HIV negative patients.

The overall mortality was 46/213 (21.6%) (Table 4), rising to 50% amongst patients with severe sepsis. Most deaths occurred within the first 10 days. KM survival analysis demonstrated a similar mortality among severe sepsis cases in the first five days of admission between HIV positive and negative patients, with worse outcomes subsequent to this in the HIV infected subset (Fig. 2). The mortality amongst adults with severe sepsis co-infected with HIV was 53.8% (14/26) compared to 33.3% (2/6) in those who were HIV uninfected. HIV positive sepsis patients who had started ART within the last 90 days had a higher hazard of death than those on ART for greater than 90 days (Hazard ratio = 2.6, 95% CI [1.01–6.8], *p* = 0.049). A statistically significant difference in death rates according to duration on ART was not found on analysis of the severe sepsis subset alone which may relate to the small numbers of patients. Rates of severe sepsis or mortality did not differ significantly between those on ART less than 90 days and ART naive HIV positive patients, odds ratio = 0.9, 95% CI (0.3–2.6), *p* = 0.832 and HR = 0.7, 95% CI (0.3–1.7), *p* = 0.471 respectively. Conversely, treatment with ART for more than 90 days was associated with a reduced mortality to the extent that no significant difference remained when compared to HIV negative patients (HR = 0.8, 95% CI [0.3–2.4], *p* = 0.735).

Independent predictors of severe sepsis included male sex, decreased temperature, reduced GCS, reduced haemoglobin and increased respiratory rate (Table 5). Risk of death increased significantly with increasing number of sepsis criteria present, although this association did not hold for the severe sepsis criteria. On multivariate analysis, independent risk factors for death were reduced systolic blood pressure, reduced percentage oxygen saturation, lower haemoglobin and male sex (Table 6).

## Discussion

Bloodstream infection (BSI) is known to account for a considerable burden of morbidity and mortality in resource constrained, high HIV prevalence countries.^22^ However successful treatment of BSI requires an ability to immediately recognise and empirically treat the clinical syndromes of sepsis and severe sepsis. Testing of interventions aimed at early empirical management of sepsis is limited by a lack of validated criteria for making syndromic diagnosis of sepsis in these settings. Here we show that using a modification of international criteria,^6,8,23,24^ amongst adults with a clinical suspicion of severe infection, patients with a diagnosis of sepsis, and those at highest risk of death, can be identified. Reduced systolic blood pressure, reduced percentage oxygen saturation and a low haemoglobin were independent risk factors for death amongst the whole cohort, with male sex, decreased temperature, reduced GCS, reduced haemoglobin and increased respiratory rate being predictive of severe sepsis; prospective validation of these factors, which are line with observations made in a similar adult population in Uganda,^4^ may enable development of a locally applicable risk stratification tool.

We found that the mortality from severe sepsis was 50% compared with 17% for patients with sepsis. This mortality is higher than that reported from industrialised countries where critically ill patients are predominantly treated in intensive care units^12,25^ and is likely to have been even higher had we had access to outcome data at 30 days.^4^ Almost two thirds of the cohort presented with sepsis as their index presentation for HIV and notably, most of the patients with known positive HIV status were already on ART. Having been on ART for more than 90 days reduced mortality by almost two thirds compared with those who had commenced treatment more recently; of these, two thirds of those with sepsis, and three quarters of those with severe sepsis died, a mortality rate similar to that of their ART naive counterparts. This is likely to have a considerable impact in Malawi and in similar countries. Malawi has an estimated national HIV seroprevalence of 12%^26^ and since 2004, has benefitted from large scale antiretroviral therapy (ART) rollout, together with widespread implementation of cotrimoxazole prophylaxis.^27^ By the end of 2009, 271,105 HIV-infected individuals had been registered on the national ART programme of whom 73% remained alive and on treatment.^28^ Nonetheless our data suggest that in addition to the time needed for immune restitution, sepsis contribute to this adverse outcome, in addition to intercurrent malnutrition and anaemia,^29^ and unmasking of underlying TB.^30^ Previous studies in Malawi have demonstrated superadded bacterial infections to be a major cause of death during the initial two months of TB therapy and that septic patients with *Mycobacterium tuberculosis* bacteraemia have a high in-hospital mortality.^31–34^Thus mycobacterial infection may have contributed to a poor outcome in our cohort but were unable to determine this with any certainty.

Optimal management of these septic HIV positive patients therefore requires specific interventions including early initiation of ART, – real-time diagnosis of both pulmonary and disseminated TB, nutritional support, and prophylaxis against opportunistic infection. Our data further emphasise the survival benefits of HIV diagnosis and introduction of ART at the earliest appropriate opportunity.

Together with previous studies,^4^ our data show that well-recognised diagnostic criteria for severe sepsis identify patients at high risk of death. Such criteria may have benefit as inclusion criteria for clinical trials of simple cost-effective interventions based on WHO guidelines. Only thrombocytopenia as a marker of severe sepsis in the context of HIV^15,16^ and falling CD4 counts^35^ are likely to have limited utility. Although guidelines developed in high income countries define the standard of care for severe sepsis patients, these do not address the operational realities of providing health care with constrained resources or use evidence from these settings.^11^ African hospitals are less likely to have ICUs, appropriate drugs, access to supplemental oxygen and monitoring equipment and or adequate human resources,^36^ and the need to develop solutions pertinent to such clinical settings is pressing. Furthermore, the role of fluid replacement and fluid resuscitation in the management of African children with sepsis has undergone considerable scrutiny following the unexpected finding of increased mortality in children with sepsis receiving bolus fluids.^37^ In a prospective adult severe sepsis intervention study conducted at two Ugandan hospitals, patients receiving early monitored management had a 30% decreased risk of 30-day mortality compared with historical control patients receiving standard management.^21^ There is therefore an urgent need to evaluate currently available interventions, including fluid resuscitation, in the management of sepsis in African adults, ideally as part of a goal-directed bundle of care.^21,38,39^

### Limitations

There are several limitations to our data. This study was undertaken at a single centre but we maintain that based on comparability with the limited number of similar studies from the region (which have largely been single centre) it is generalizable to other high HIV prevalence settings. Assessment of outcome was only possible in-hospital rather than the standard 30-day follow-up into the community, which is likely to have led to an underestimate of mortality. We had limited access to laboratory investigations including inflammatory markers (e.g. no access to CRP or procalcitonin), CD4 counts and more specific microbiological tests such as cryptococcal antigen, toxoplasma serology or virology diagnostics. Smear-positive tuberculosis should be apparent using available tests such as sputum microscopy and chest radiography,^32^ but in acutely unwell patients diagnosis remains challenging and mycobacterial cultures were not performed. Additionally, although the sepsis and severe sepsis criteria used identified adults with a high risk of death, we did not have access to invasive or non-invasive monitoring techniques to physiologically confirm these diagnoses. Finally, patients were enrolled with a clinical suspicion of severe infection and we did not screen all medical admissions for sepsis criteria. We therefore may have missed potentially eligible subjects. Subsequent studies should take this into consideration.

### Conclusions and recommendations

This study confirms that severe sepsis in a sub-Saharan Africa setting has high mortality amongst both HIV-infected and uninfected adults. Establishment on ART confers significant survival benefit in the HIV positive subset, and the high mortality among patients on ART for less than three months underscores the importance of vigilant clinical follow-up among this group. Patients at risk of death can be identified using simple, objectively measurable criteria, which following validation amongst other populations can be used to standardise multi-site interventional trials of sepsis bundles in resource-poor settings. These will enable the formulation of appropriate evidenced-based local guidelines and clinical trials for the management of critically ill adults in sub-Saharan Africa.

## Figures and Tables

**Table 1:** Criteria used for the diagnosis of a) Sepsis and b) Severe Sepsis.

a) Sepsis criteria (at least 2 present)
Temperature <35 °C or >38.3 °C
Respiratory rate >20 breaths/minute
Pulse >90 beats/minute
White cell count >12 or <4 × 10^9^ cells/l
Altered mental state

b) Severe sepsis criteria (at least 2 sepsis criteria and either systolic blood pressure <90 mmHg, or at least 2 severe sepsis criteria present)
Thrombocytopenia <100,000 × 10^9^ cells/l (if HIV neg)^a^
Oxygen saturations in room air (<90% on room air)
Systolic blood pressure <90 mmHg
Reduced capillary refill (>2 s)

a Thrombocytopenia is common in HIV infected adults.

**Table 2:** General demographics, HIV features and clinical characteristics of Malawian adults with suspected sepsis.

	All patients *n* = 213	Sepsis *n* = 181	Severe sepsis *n* = 32	p*-value
**General demographics:**				
Sex (Female), *n* (%)	126 (59.2)	105 (58.0)	21 (65.6)	0.272
Age (years), median (IQR)	30 (25–39)	33 (25–39)	33 (25–39)	0.100^a^
**HIV features:**				
Number HIV infected, *n* (%) of HIV positive subset,	161 (75.6)	135 (74.6)	26 (81.3)	0.286
Number aware of prior HIV diagnosis, *n* (%)	88 (54.7)	70 (51.9)	18 (69.2)	0.08
Number on ART, *n* (%)	70 (43.5)	58 (43.0)	12 (46.2)	0.464
On ART <90 days, *n* (%)	28 (21.7)	21 (15.6)	7 (26.9)	0.133
**Clinical characteristics:**				
Temp (^°^C), median (IQR)^a^	38.8 (38.1–39.4)	38.8 (38.1–39.4)	38.2 (38.0–39.2)	0.056
Pulse rate (bpm), median (IQR)^a^	105 (96–120)	104 (92–120)	120 (104–140)	<0.001^a^
Respiratory rate (/min),median (IQR)^a^	26 (22–32)	26 (22–32)	30 (24–38)	0.100^a^
SBP in mmHg, median (IQR)^a^	110 (100–120)	110 (100–120)	82.5 (80–105)	<0.001
Glasgow Coma Scale, Mean (sd)^b^	14.6 (2.0)	14.7 (1.5)	14.2 (2.0)	0.05

Oxygen saturations, median (IQR)^a^	93 (90–96)	94 (90–96)	89 (82–93)	<0.001

P*-value: fisher's exact *p*-value has been reported for associations between categorical variables and diagnosis.

a Man-Whitney *U* test was performed.

b *t*-test was performed.

**Table 3:** Laboratory characteristics of Malawian adults with suspected sepsis.

Haematology	All patients *n* = 213	Sepsis *n* = 181	Severe sepsis *n* = 32	*p*-value
Haemoglobin (g/dl), median (IQR)	10.1(7.4–11.8)	10.4(7.8–11.8)	8.8(6.1–11.3)	0.840
WBC x10^9^/l, median (IQR)	6.1(3.8–10.1)	6.1(3.8–10.1)	5.9(4.2–10.9)	0.990

Blood culture^a^	*n*/*N* (%)
Non Typhoidal Salmonella	16/38 (42.1)
*Streptococcus pneumoniae*	9/38 (23.7)
*Cryptococcus neoformans*	3/38 (7.9)
*Escherichia coli*	2/38 (5.3)
*Klebsiella* spp	2/38 (5.3)
*Staphylococcus aureus*	1/38 (2.6)
Contaminants	5/38 (13.2)

CSF culture^a^	*n*/*N* (%)
*Streptococcus pneumoniae*	4/41(9.8)
*Cryptococcus neoformans*	3/41(7.3)

a 1 patient grew Streptococcus pneumoniae in both blood and csf cultures.

**Table 4:** In-hospital mortality amongst Malawian adults with suspected sepsis.

	All patients *n* = 213	Sepsis *n* = 181	Severe sepsis *n* = 32	a*p*-value
Total Deaths, *n* (%)	46/213 (21.6)	30/181 (16.6)	16/32 (50.0)	<0.001
Deaths by 48 h, *n* (%)	13/213 (6.1)	8/181 (4.4)	5/32 (15.6)	0.014
Mortality HIV neg, *n* (%)	7/52 (13.5)	5/46 (10.9)	2/6 (33.3)	0.180
Mortality HIV+, not on ART, *n* (%)	21/91 (23.1)	13/77 (16.9)	8/14 (57.1)	0.003
Mortality HIV+, on ART >90 days, *n* (%)	7/42 (16.7)	6/37 (16.2)	1/5 (20.0)	0.618
Mortality HIV+, on ART <90 days, *n* (%)	11/28 (39.3)	6/21 (28.6)	5/7 (71.4)	0.060

a Fishers exact test.

**Table 5:** Multivariate analysis: independent predictors of diagnosis of severe sepsis.

Risk factor	Odds ratio for severe sepsis	95% confidence interval
Male sex	2.9	1.2–7.1
Temperature <38 °C	3.2	1.2–8.3
Glasgow Coma Scale<15	14.2	3.4–60.3
Hb (g/dl) (per unit decrease)	1.3	1.1–1.4
Respiratory rate > 20 breaths/min)	7.0	1.4–34.8

**Table 6:** Multivariate analysis: Independent predictors of inpatient mortality for all patients with sepsis.

Risk factor	Odds ratio	95% confidence interval
Male sex	2.2	1.04^a^–4.5
SpO_2_<90% on room air	2.5	1.1–5.4
Systolic BP < 90 mmHg	6.0	2.0–18.5
Hb g/dl	0.8	0.7–0.9

a Not rounded because it is significant, and inference changes when rounded.

## Abbreviations

ART: antiretroviral therapy
BSI: bloodstream infection
CRP: C-reactive protein
CSF: cerebrospinal fluid
GCS: Glasgow coma score
HIV: human immunodeficiency virus
HR: Hazard ratio
ICU: intensive care unit
KM: Kaplan–Meier
NTS: non typhoidal salmonellae
OR: odds ratio
QECH: Queen Elizabeth central hospital
SBP: systolic blood pressure
SIRS: systemic inflammatory response syndrome
SSA: sub-Saharan Africa
TB: tuberculosis
WHO: World Health Organisation
